# Influence of Composition Modification of Basalt Fiber-Reinforced Polymer Bars on Alkali Resistance

**DOI:** 10.3390/polym18050637

**Published:** 2026-03-05

**Authors:** Andrzej Garbacz, Maria Włodarczyk, Grzegorz Banasiak

**Affiliations:** Faculty of Civil Engineering, Warsaw University of Technology, Al. Armii Ludowej 16, 00-637 Warsaw, Poland; maria.wlodarczyk@pw.edu.pl (M.W.); grzegorz.banasiak@yahoo.com (G.B.)

**Keywords:** BFRP bars, hybrid FRP, alkali resistance, tensile strength, shear strength

## Abstract

The application of fiber-reinforced polymer bars has been considered an alternative for the non-metallic reinforcement of concrete structures. Basalt fiber-reinforced polymer (BFRP) is a new composite used to reinforce concrete structures. However, the main drawback of BFRP is its low modulus of elasticity. Therefore, hybrid reinforced fiber polymers, in which carbon fibers replace part of the basalt fibers, might be considered as a relatively “simple” modification that can increase the modulus of elasticity. The literature data suggest that modification of the epoxy matrix with nanosilica particles can positively influence resistance to high temperatures. Besides the mechanical characteristics of FRPs, the evaluation of alkali resistance is necessary for technical approval for construction applications. This paper focuses on testing the alkali resistance of basalt fiber-reinforced polymer (BFRP) bars and its modification through the partial substitution of basalt fibers with carbon fibers (HFRP) and the addition of nanosilica to the epoxy binder (nHFRP). The alkali resistance was tested based on the most common method described in ACI report 440.3R-04—part B6. This method consists of three procedures carried out at 60 °C on the specimens immersed in an alkaline solution, both with and without load. The changes in the mass and tensile strength of the bars are examined after 1, 2, 3, 4, and 6 months. The test procedures are time-consuming and expensive, particularly Procedures B (in alkaline solution) and C (in concrete cover), in which longitudinal tested specimens must be immersed in alkaline solution and subjected to constant strain at an elevated temperature for a 6-month period. Therefore, this study proposes a test setup to achieve a less time-consuming and cheaper assessment of the alkali resistance of FRP bars. Additionally, the usefulness of the shear strength test for the evaluation of alkali resistance of FRP bars is also discussed. The results (Procedure A) indicate that modification of the composition of BFRP did not decrease the resistance to the alkaline environment in the case of HFRP (5% lower than in the case of BFRP). Under the same conditions, the decrease in the tensile strength of nHFRP was 40% higher than in the case of BFRP. This indicates that additional modification of the composition by adding nanosilica to the epoxy binder did not provide the expected stability of tensile properties at elevated temperatures. The results of the evaluation of alkali resistance according to Procedure B show that the device proposed for maintaining constant strain during the seasoning is promising. At this stage, the device makes it possible to conduct the tests at ambient temperature and yields a significantly lower decrease in tensile strength (10–14%) after 6 months, demonstrating a significant effect of temperature on the results of the FRP alkali resistance test.

## 1. Introduction

The maintenance and repair of infrastructure represent a key challenge in modern civilization [1]. The corrosion of steel reinforcement due to the carbonatization of concrete covers, chloride-induced corrosion, and the destruction caused by freeze–thaw cycles are the most common causes of damage to concrete structures during their service [1,2]. Recently, fiber-reinforced polymer (FRP) bars have been considered as an alternative non-metallic reinforcement for concrete structures due to their superior chemical resistance, especially in the presence of chloride ions [3,4,5,6].

FRPs are composite materials consisting of a polymer matrix reinforced with fibers, where approx. 70–80% of the volume comprises fibers, while the remaining 20–30% is a polymer matrix. FRPs are produced using pultrusion techniques. Different types of fiber are used, including glass, aramid, carbon, and basalt, as well as different types of matrix, such as epoxy, vinyl, and polyurethane resins. Various fiber winding techniques are commonly used to improve the adhesion of a composite to the concrete cover, including parallel or cross-winding [4]. FRP bars have several advantages over steel reinforcement bars, including comparable or superior physical and chemical properties, a thermal expansion coefficient similar to that of concrete, and resistance to corrosion, radiation, UV light, and vibrations. Their weight is approx. 10 times lower than traditional steel bars [3].

Among the types of FRP bar, GFRP bars are the most popular due to their relatively low cost. However, they are brittle and have insufficient resistance to corrosive environments [7,8,9]. As a result, researchers are exploring alternative FRP composites such as basalt fiber FRP bars, which have shown better resistance to alkaline environments. The use of hybrid FRP reinforcing bars, combining different fibers, is also being investigated as a potential solution to improve the elastic modulus of these materials [5]. Research on the properties of basalt fiber composite bars has been carried out worldwide since the 1990s [5,7,10].

Table 1 summarizes the values of the basic strength properties for reinforcing bars, made of steel (reference values) and FRP bars that differ in the type of reinforcing fibers: GFRP (glass fiber-reinforced polymer), CFRP (carbon fiber-reinforced polymer), AFRP (aramid fiber-reinforced polymer), and BFRP (basalt fiber-reinforced polymer). The variations in the properties for the given FRP type, presented in Table 1, result from the nature of the fibers, their content, the resin type, and the technological conditions of pultrusion.

The durability of FRP composites depends largely on the corrosion resistance of the resin used, while the fibers used are mainly responsible for the mechanical properties. The resin also plays a role in bonding, filling, and protection, and distributes stress [11]. Corrosion processes always begin with damage to the resin cover and a weakening of its adhesion to the fibers. The deterioration of FRP composites accelerates when the material is subjected to stress during seasoning [12,13]. When an FRP bar is subjected to a load, microcracks can appear on the resin surface, through which corrosive agents penetrate the fibers. In addition, immersion of the FRP composite in a corrosive environment (water, alkaline solution) causes its absorption by the resin, which in turn causes delamination and further crack formation, resulting in an increase in corrosion effects as the corrosive agents can easily penetrate into the fibers [14]. There are many studies in the literature on the deterioration of FRP bars e.g., [8,11,14,15,16,17,18] related to the effects of alkaline environments, temperature (freeze–thaw cycles and high temperatures), fluid absorption and its effect on physical–mechanical properties, creep, fatigue, seasoning, moisture and aqueous solutions, long-term loading, UV radiation (which is less important in the case of reinforced FRP), and fire. The variety of potential causes of failure of FRP bars can be detected in alkali resistance tests.

In order to illustrate the impact of alkaline environments, temperature, and the time of seasoning on the resistance of various types of FRP bars, the results of selected experimental studies (in similar alkaline environments) available in the literature are presented in Table 2. These results show the complexity of the problem, and it is not possible to clearly link the variability of a retention ratio of tensile strength to the changes in temperature, time of seasoning, pH value, and stress level during seasoning. However, some authors observed a decrease in the retention ratio with an increase in temperature at the same time of seasoning. Other researchers [9,11,19,20,21,22,23] have also made similar observations for other types of environment.

The issue of durability in FRP bars is more complicated because of the following [6,15]:

− Degradation processes involve not only the fibers themselves, but also the resin and the interactions between them.
− Different types of fibers behave differently when exposed to various temperatures, environmental conditions, and rheological effects.
− Material degradation more broadly has differential effects on individual strength properties, e.g., the effects of moisture and UV on the tensile strength (stresses acting along the fibers) of FRP materials with vinylester resin (generally more durable than others) are negligible compared to their effects on flexural strength and stiffness in the transverse direction.

The complexity of FRP durability provided above clearly indicates a necessity for standardized assessments of the properties of FRPs, including alkali resistance.

Recently, basalt fibers have been considered as an economical substitution of glass fibers in FRPs. They are resistant to high (even up to 700 °C [28]) and low temperatures; exhibit low water absorption and high corrosion resistance, including acid and alkali resistance; high strength and resistance to fatigue effects; resistance to ultraviolet and electromagnetic fields; and resistance to moisture and air [29]. Studies show [30] that basalt fibers have higher corrosion resistance (especially in acidic environments) than glass fibers and lower weight loss. However, both types of fibers show lower durability compared to carbon fibers [5]. Basalt fibers work well with almost any type of resin: epoxy, polyester, phenolic, melanin, acrylic, and polyurethane [28], and vinyl ester resin, which shows the best resistance to harmful effects of moisture or ultraviolet [15,31]. Studies have shown that BFRP composites with epoxy resin have higher initial strength properties and a lower relative decrease after seasoning in an alkaline environment [19]. This property is especially important in the case of fresh concrete and this is why it is essential to assess alkali resistance.

New modifications of the structure of FRP composites are still being implemented, which may include the composite fibers, their type and arrangement, and matrix additions. One example of the modifications related to composite fibers are hybrid fiber-reinforced polymer (HFRP) composites. In hybrid composites, different fibers are used in the cross-section of the composite. For example, in BFRPs, a part of the basalt fibers can be substituted with carbon fibers to increase the stiffness of BFRP bars as well as the modulus and strength [4]. The fatigue performance of glass-reinforced polymers (GFRPs) can also be improved by adding carbon fibers. The high performance offered by hybrid composite materials have led to their continued development [1,3].

This paper focuses on the alkali resistance of BFRP bars and their modification with carbon fibers (HFRP bars) and additionally nanosilica (nHFRP bars). This research was part of a bigger project conducted at Warsaw University of Technology and Białystok University of Technology in cooperation with an industry partner that was planning to start the production of various commercial BFRP and HFRP bars. Besides mechanical characteristics, the evaluation of alkali resistance is necessary for technical approval for application in construction. The most common method applied to test the alkaline resistance of FRPs is described in the report of the American Concrete Institute ACI 440.3R-04—part B6 [32]. This test method described by the ACI is lengthy (the test schedule for one batch is at least 200 days), complicated (involving simultaneous immersion, stretching, and heating of the specimen to 60 °C for six months), and expensive. This is likely why it is far more common in the literature to find the changes in mechanical properties of composites being tested using simplified methods.

The three main goals of the research presented in this paper were as follows:

− To investigate the effect of modifying the composition of BFRP bars through hybridization, i.e., partially replacing the basalt fibers with carbon fibers (HFRP bars) and with an additional admixture of resin with nanosilica (nHFRP), on alkali resistance.
− To propose a test setup for a less time-consuming and cheaper assessment of alkali resistance of FRP bars.
− To analyze the usefulness of the shear strength test to evaluate the alkali resistance of FRP bars.

## 2. Materials and Methods

### 2.1. Materials

Extensive research on the use of BFRP bars for RC structures is being conducted at Warsaw University of Technology (Poland) and Białystok University of Technology in cooperation with an industrial partner. The research aims to investigate the structural performance of various FRP bars for RC structures and determine the most reliable FRP bar type that can balance safety and cost parameters [33]. Experimental testing of the strength performance of concrete beams reinforced with BFRP bars described in [22,34] shows that the deflections of beams reinforced with BFRP bars are approximately five times higher than those of beams reinforced with steel bars; this may cause concrete crushing in the compression zone before a balanced ratio between reinforcement and concrete strength characteristics can be obtained. The reason for such deflections is the relatively low modulus of elasticity comparing to steel bars. To improve BFRP bars, hybridization of BFRP was proposed, where part of the basalt fibers were substituted by carbon fibers. PHFRP bars are processed in the same way a BFRP bars, in which a certain amount of the basalt roving is replaced with carbon roving during the pultrusion process. In addition, two different adhesive agents were used for the HFRP bars: conventional epoxy resin and epoxy resin modified with nanosilica (nHFRP) to improve the properties of the resin at elevated temperature.

The effect of the ratio of carbon to basalt fibers and the influence of their location on the mechanical properties of hybrid composites are discussed in previous companion papers [10,35], along with a detailed and extended description of the characteristics of the bars and their configurations. Several technological challenges were encountered while placing carbon fibers in the near-to-surface region, including increased heterogeneity at fiber locations and local scorching of bars caused by temperature changes. Thus, carbon fibers are most effective in the core region of HFRP bars, where a carbon/basalt ratio of 1:4 is assumed (i.e., 16% carbon fibers, 64% basalt fibers, and 20% epoxy resin by volume).

The subjects of the alkali resistance tests were BFRP bars and two types of HFRP (basalt–carbon) bars, ∅8 mm in nominal diameter, with epoxy resin as the matrix:

− BFRP: basalt fibers 100%;
− HFRP: basalt fibers 75%, carbon fibers 25%;
− nHFRP: basalt fibers 75%, carbon fibers 25%, and epoxy resin modified with the addition of nanosilica (3% by weight).

The epoxy system 1300, developed by Ciech Sarzyna S.A. (Sarzyna, Poland), was used as the matrix for the hybrid composite bars. It consists of the following components:

− Component A—Epidian 1300 low-molecular-weight epoxy resin (derived from bisphenol A and epichlorohydrin);
− Component B—waterless hardener 1300;
− Component C—1300 accelerator in the form of a tertiary aliphatic amine, used as an epoxy resin crosslinking agent;
− Component D—1300 modifier (polypropylene glycol diglycidyl ether), used as an active diluent to increase the flexibility of the resin.

The 1300 epoxy system was developed for the production of structural composites using the pultrusion method. According to the manufacturer’s recommendations, the individual components were mixed using a mechanical stirrer at an A:B:C:D ratio of 100:70:5:7 (by weight) and the glass temperature of this epoxy system was 60 °C.

**Figure 1:**
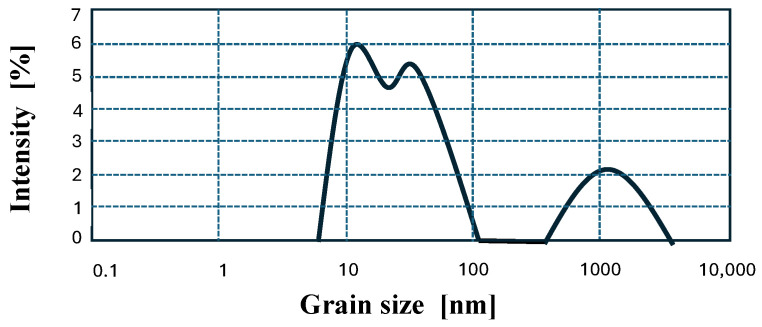
Average particle size distribution of nanosilica particles used to modify the epoxy resin used as a matrix in nHFRP bars (based on 3 independent measurements).

In addition, a second type of epoxy matrix was prepared with the addition of commercial nanosilica. A sol with nanosilica particles with a concentration of 25 ÷ 30% by weight was used. The particle size of the nanosilica used as a modifier was determined using a Malvern apparatus (Malvern, UK). The average size of the nanosilica used was 24.4 nm, with two distinct fractions: a finer one with a peak at 30 nm (approximately 80%) and a coarser one with a peak at 1270 nm (approximately 20%), as shown in Figure 1.

Prior to the tests, the geometrical characteristics of the test bars were characterized and the strength properties (tensile and shear) of the control specimens were investigated (Table 3). The substation of part of basalt fibers with carbon fibers in the case of HFRP favorably increased the elasticity modulus and tensile strength values. The details are given in [13].

Additionally, observations were performed of the microstructure homogeneity of the tested FRP bars. The microstructures images were registered for a polished cross-section with a scanning electron microscope (ZEISS LEO 1430, Carl Zeiss AG, Oberkochen, Germany). Table 4 shows examples of the typical morphology of the cross-section of the tested BFRP, HFRP, and nHFRP bars at different magnifications.

In general, both the basalt and carbon fibers in the tested FRP types show heterogenous distributions. In the case of nHFRP bars, nonhomogeneous distributions of larger nanosilica particles and their agglomeration were observed. However, despite the microstructure heterogeneity of all types of tested FRP bars, relatively low scattering of mechanical properties was obtained. The coefficient of variation was no higher than 4.6% in all tested properties. The highest scattering of values was observed for the mechanical properties of HFRP bars.

### 2.2. Alkali Resistance Testing of FRP Bars

Various test methods have been developed to evaluate the alkali resistance of FRP composites, including exposure to alkaline solutions and direct contact with concrete. Numerous models of FRP bar durability can be found in the literature, ranging from statistical molecular models and linear elasticity models to current attempts at modeling by measuring strength loss during seasoning and water absorption models according to diffusion laws [28,34]. The degradation of FRP bars is not constant over time (it progresses primarily in the first period—around 1000 h), and durability studies are usually conducted using the assumptions of the Arrhenius equation. This equation determines the proportionality between the rate of the chemical process and the temperature of the reaction, which allows for an accelerated approximation of the degradation effects of materials subjected to seasoning under various aggressive conditions [34].

In testing the chemical resistance of bars used for reinforcing concrete structures, the greatest emphasis is placed on testing resistance to a strongly alkaline environment, simulating the effects of concrete lagging on the bar. The Eurocodes do not propose any specific methods for testing the physicochemical properties of composite bars. However, a method for testing these properties is described in the American Concrete Institute report ACI 440.3R [32].

The alkali test method described in ACI 440.3R part B6 consists of 3 procedures carried out at 60 °C, each with a different load applied to the tested elements. Samples of bars immersed in an alkaline solution with or without load are examined after 1, 2, 3, 4, and 6 months. The changes in the mass and tensile strength of the bars after each period are measured. Samples are seasoned for 40 h at 23 °C and 50% relative humidity before the test. The details of the ACI test procedure are listed below:

− Procedure A: A total of 25 samples for each size and type and 5 samples as a control group are immersed in an alkaline solution: 118.5 g of Ca(OH)2, 0.9 g of NaOH, 4.2 g of KOH, and 1 L distilled water. The pH of the solution is maintained at 12.6 to 13 and the container with the samples should be sealed. The test is carried out at 60 °C.
− Procedure B: A total of 25 samples for each size and type and 5 samples as a control group are immersed in an alkaline solution: 118.5 g of Ca(OH)2, 0.9 g of NaOH, 4.2 g of KOH, and 1 L distilled water. The pH of the solution is maintained at 12.6 to 13 and the container with the samples should be sealed. The procedure is carried out at 60 °C. During the test, the samples are subjected to a given constant tensile load causing a deformation at the level of 2‰.
− Procedure C: A total of 25 samples for each size and 5 samples as a control group are embedded in concrete (cylinder concrete samples with a diameter of 150 mm and a height of 200 mm, or minimum 40 sample diameters). It is recommended that the same constant tensile load as Procedure B is applied.

Five ∅150 × 300 mm concrete samples should be tested for compressive strength after 28 days for reference according to ASTM C39. The procedure is carried out at 60 °C and the concreted section should be kept moist at all times.

In each procedure, a change in mass and tensile strength is tested. The pH level of the alkali solution and the temperature are checked at the beginning, during, and at the end of the test. The external appearance of the tested bars is also evaluated (color, surface, shape, and cross-section under the microscope).

In this work, Procedure A as well as modified Procedures B and C of the ACI 440.3R part B6 were used:

− Procedure A: No changes were applied. The samples were immersed in an alkaline solution as described in the ACI method and kept at 60 °C for 1, 2, 3, 4, and 6 months.
− Procedure B: The tensile load was applied to the samples according to the ACI guideline, and the bars were immersed in an alkaline solution. In this case, the samples were kept at 23 °C and the tensile strength was tested after 1, 2, 3, and 6 months;
− Procedure C: The samples were embedded in concrete, a tensile load was applied to the samples according to the ACI guideline, and the bars were constantly moisturized. The testing conditions were the same as in the modified Procedure B; however, the tensile strength was instead determined after 1, 2, and 3 months.

In all procedures, the alkaline solution was prepared in proper volume to maintain the recommended proportion: 118.5 g of Ca(OH)_2_, 0.9 g of NaOH, and 4.2 g of KOH were solved in 1 L of distilled water.

For Procedure A, specimens of 1420 mm in length were prepared (25 specimens for each type of bar; 75 specimens in total). Five samples of each type of bar were allocated for each of the five test periods. After seasoning, the specimens were restrained in ∅42.5 steel tubes in a special high-strength mortar.

For Procedure B, bars of 1610 mm in length were prepared (14 specimens for each bar type; 42 bars in total). A total of 3 samples of each type of bar were studied for each of the three test periods of 1, 2, and 3 months, while 5 samples were selected for the 6-month period. First, one-sided anchorage of all bars was performed. Then, pre-cut PE32 mm pipes with a length of approx. 330 mm were placed on the specimens from the unanchored side, ending on both sides with plugs with 8 mm diameter holes. Anchoring of the other sides was performed and the specimens were placed in the device, applying a constant strain load of 2‰, as recommended in the ACI report. The bottom plug was sealed, an alkaline solution with the same structural formula as for Procedure A was poured into the PE pipe, and the top plug was sealed. After seasoning, the PE tubes were emptied, the specimens were removed from the constant strain device, the PE tubes were removed, and finally the bars were dried, cleaned, and placed in the testing machine.

For Procedure C, bars of 1610 mm in length were prepared (9 specimens for each type of bar; 27 bars in total). Three specimens of each type of bar were allocated for each of the three test periods. Prior to testing, the specimens were weighed and the half-meter sections at the ends were restrained in 42.5 mm diameter steel tubes in special high-strength mortar. Pre-cut tubes of 150 mm in diameter and approximately 33 cm in length were then applied. Seals were made from the bottom and concrete was poured into the PE150 mm pipes according to a previously developed recipe. The specimens were then placed in the device, applying a constant load causing a deformation of 2‰. After seasoning, the specimens were removed from the constant strain device, the PE bars were removed, and the specimens were placed in the testing machine.

Three 150 × 150 × 150 mm concrete specimens were also prepared to test the compressive strength of the concrete needed for Procedure C.

The shear strength tests on FRP bars were carried out according to the procedure proposed in ACI 440.3R-04 part B.4 [36]. This procedure was considered an alternative method for the assessment of the alkali resistance of FRP bars. The samples were kept at 23 °C and 50% humidity for 40 h and then seasoned in the solution: 118.5 g of Ca(OH)2, 0.9 g of NaOH, 4.2 g of KOH, and 1 L distilled water (as in the ACI method previously described). After 6 months of seasoning at 60 °C, the shear strength tests were carried out. For the shear strength test, 5 specimens were prepared for each type of bar, each with a length of 300 mm.

### 2.3. Proposed Setup for Alkali Resistance Testing According to ACI

The proposed modifications have allowed a significant reduction in the time needed to conduct research and reduce the cost of testing for each batch. The procedure described above also allowed the effects of factors determining the durability of samples to be isolated. Thanks to the described changes, the research unit does not need to occupy the treatment chambers and testing machines for half a year. However, holders are needed to fix the samples in the testing machine and steel pipes are required to anchor the ends of the samples in all procedures. In addition to the testing machine employed to test the tensile strength, and the microscope and extensometers used to measure appropriate stress, there is also a need for the alkaline solution and concrete materials. The most disputable issue with the proposed procedure is the lack of curing at 60 °C in Procedures B and C. However, modifications to the proposed setup will provide this opportunity in the future.

**Figure 2:**
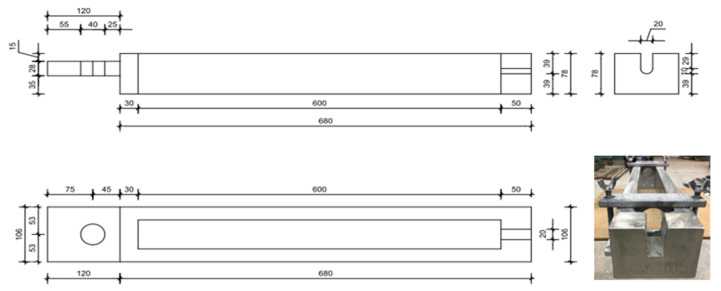
Sketches of the horizontal holders used to install the samples in a press and a photograph of a handle that allows the bars to be stretched in a horizontal press.

Sketches of the horizontal holders used for breaking bars after seasoning in the test machine are given in Figure 2.

One of the main difficulties in carrying out laboratory tests as described above is providing a load that results in a constant deformation of the test specimens. The number of specimens and the length of the seasoning period preclude the use of testing machines. On the other hand, the forces required to deform FRP bars by around 0.2% are relatively small and one can consider suspending and loading individual specimens on a common structure; however, with more than 100 specimens, such a structure also becomes large and its mobility is poor.

One of the important objectives of the authors was to develop a bar test rig according to Procedures B and C described in ACI 440.3R-04 B6. Accordingly, the authors designed a device in the form of frame allowing arbitrary (within a specified range) loading of a single bar with a specified, constant tensile force. In the proposed solution, the tensile force is transmitted through the reaction imparted by plate 2, as shown in the scheme (Figure 2). The magnitude of the force is controlled by an appropriate rotation of the nut. Increasing the pressure of the nut accordingly causes the bars to stretch, which is kept constant.

**Figure 3:**
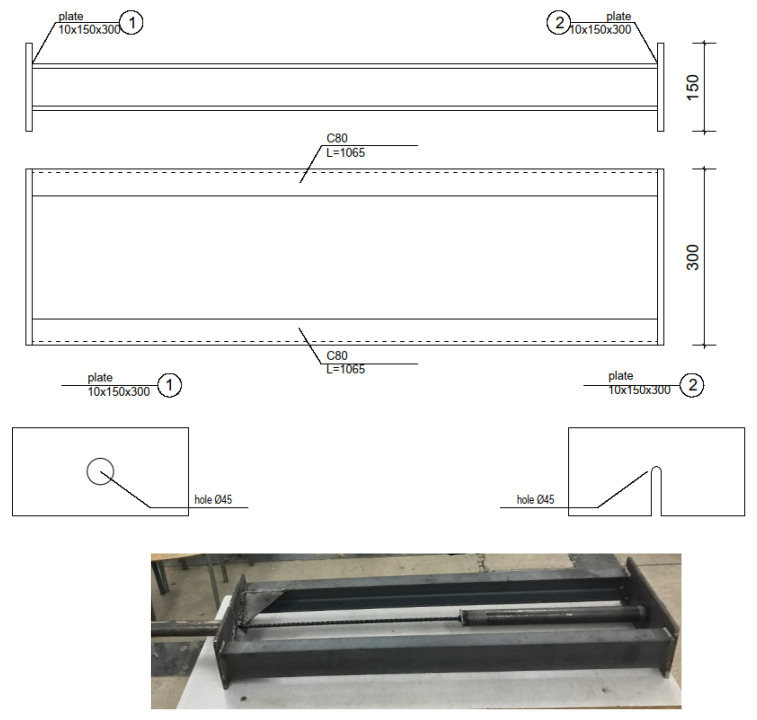
Design of the instrument allowing constant strain to be maintained during specimen seasoning (authors’ own development) and the prototype of the bar tensile frame before testing.

A preliminary test was performed for each type of bar in order to determine the torque with which the bolt must be tightened to obtain the appropriate strain in the bar. During this test, a specimen (BFRP, HFRP, and nHFRP, respectively, anchored on both sides) was placed in the prototype frame, to which an extensometer was mounted. The strain in the specimen was then gradually increased by turning the screw. When the extensometer reading indicated an elongation in the specimen corresponding to the required strain (0.02%), the tightening torque was read using a torque spanner of the appropriate scale and constantly monitored during test. Subsequent specimens were placed in the frame and tightened to the appropriate torque (Figure 3).

**Figure 4:**
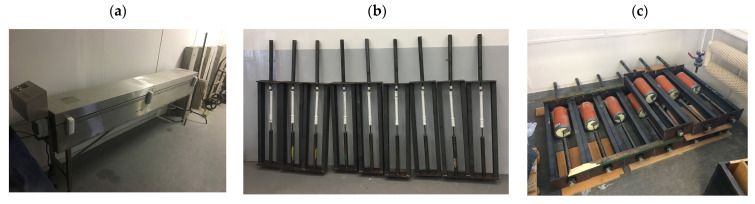
(**a**) Chamber for FRP samples seasoning according Procedure A; (**b**) samples under seasoning for Procedure B; (**c**) samples under seasoning for Procedure C.

**Figure 5:**
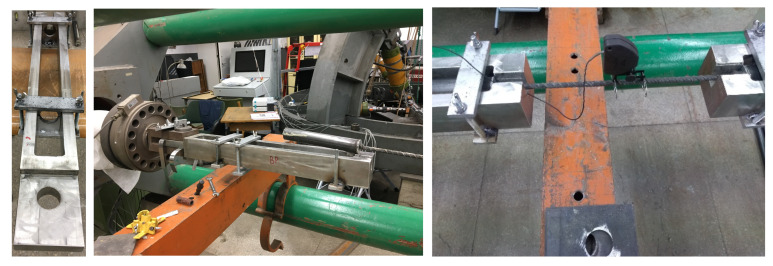
Photographs of the holder before assembly (**left**) and the holder mounted in a Walter + Bai AG laboratory testing machine with the MGCplus extensometer system (**right**).

Appropriate modification (reinforcement) of the designed structure allows, of course, the use of other, higher forces (both to obtain larger deformations and to test bars with larger diameters). The first step in the process of optimizing alkali resistance tests according to ACI 440.3R-04 B6 was testing the authors’ solution, which is a structure for maintaining constant deformation during the seasoning of specimens, whether in alkaline solution (Procedure B) or in concrete (Procedure C) according to modified procedures. In the next step, it was necessary to add a temperature factor, e.g., by using a suitable set of electric heaters and a thermostat, and the choice of materials for the solution containers and their seals needed to be revised so that they remained functional at the elevated test temperature. Figure 4 shows the FRP sample during seasoning according to 3 ACI procedures and Figure 5 shows the FRP samples in the Walter + Bai AG (Löhningen, Switzerland) horizontal testing machine (type: HZWU 2-280/140/4005) with the MGCplus extensometer system. The loading rate was equal to 15 MPa/s.

### 2.4. Shear Strength Testing

**Figure 6:**
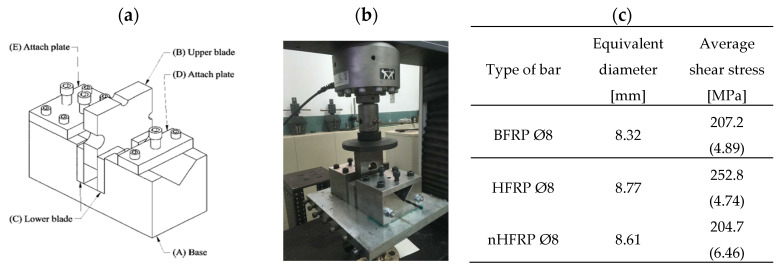
The shear strength test: (**a**) scheme of loading device according to ACI 440.3R-04, part B.4; (**b**) the testing machine with the integrated loading device; (**c**) average value of shear strength and coefficient of variation (in %) of reference FRP bars (acc. [37]).

The shear strength tests were carried out using the Instron 3382 Floor Model Universal Testing System (Instron, Norwood, MA, USA), which had a loading capacity exceeding the shear capacity of the test specimen and was calibrated according to the ACI 440.3R-04: part B.4 [36] (Figure 6). The testing machine was in the typical test setup. It consisted of a sample holder, one upper blade, and two lower blades. The shear strength testing device was constructed of steel. During the test, the bar-shaped specimen was sheared on two planes simultaneously by the blades, which were converging along faces perpendicular to the axis of the test specimen. The loading rate was 0.5 MPa/s.

## 3. Results

### 3.1. The Results of Tensile Strength Testing After Seasoning

**Figure 7:**
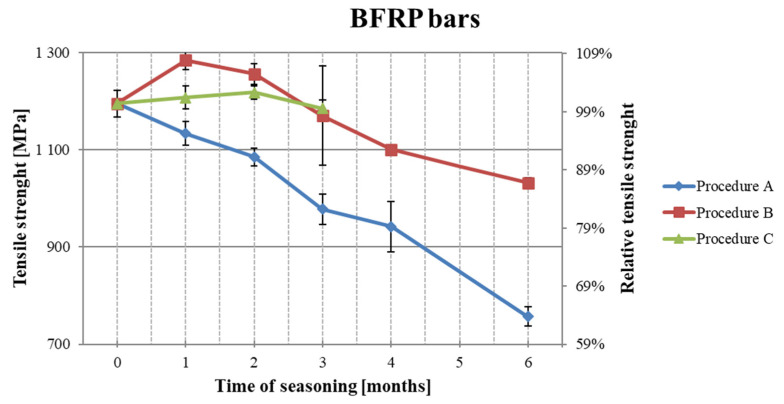
Decreases in the tensile strength of BFRP bars depending on the seasoning period.

**Figure 8:**
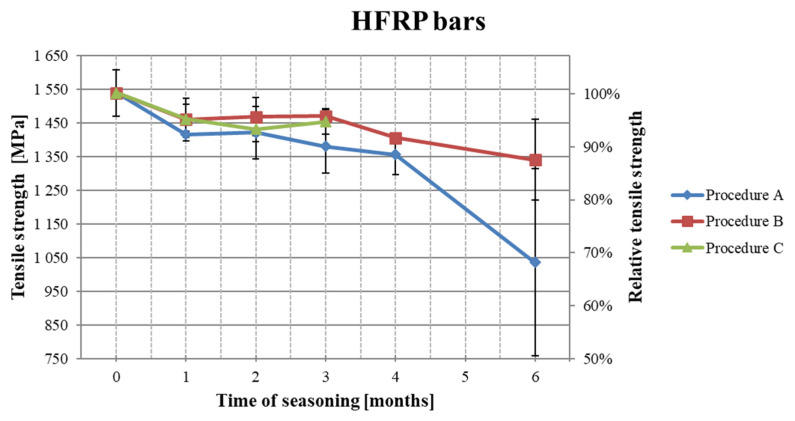
Decreases in the tensile strength of HFRP bars depending on the seasoning period.

**Figure 9:**
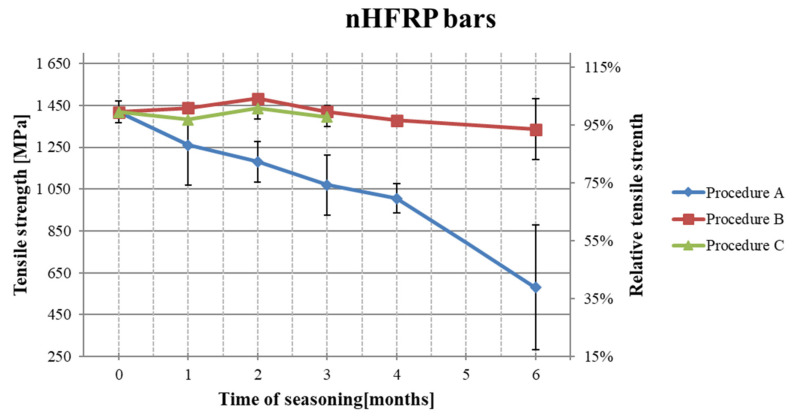
Decreases in the tensile strength of nHFRP bars depending on the seasoning period.

**Figure 10:**
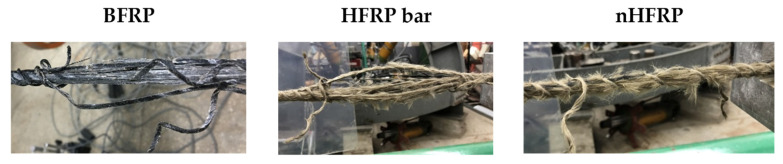
Typical failure modes after 6 months of seasoning according to Procedure A.

**Figure 11:**
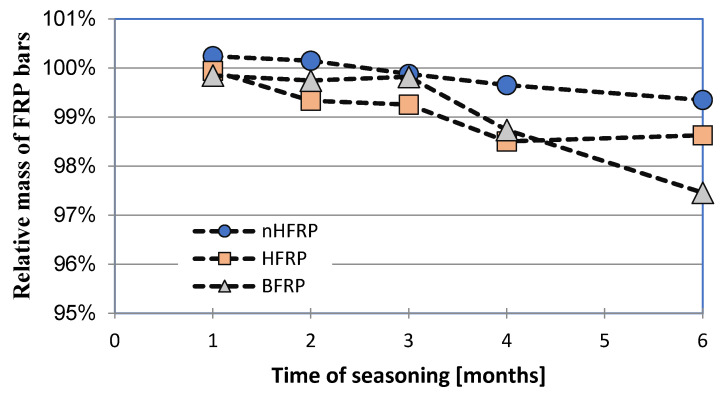
Relative decreases in the weights of the bars in the procedure depending on the time of seasoning—Procedure A.

The results of the tensile strength tests after the subsequent period of seasoning are shown in Table 5. Figure 7, Figure 8 and Figure 9 present the decrease in the tensile strength of the three types of FRP bars after seasoning in the alkaline environment. Each data point represents the mean value of three or five samples. Five samples were tested for pre-season reference values and for Procedure A. In the case of Procedures B and C, three samples were tested, taking into account the low scattering of the reference values for mechanical properties, i.e., a low coefficient of variation. All types of samples (BFRP, HFRP, and nHFRP) exhibited elastic properties up to a rapid breakage. The breakage most often occurred in the fibers and less frequently in the entire cross-section of the bar. The most frequent and the most specific breaks are shown in Figure 10. The changes in mass after 6 months of seasoning (Figure 11) were relatively low; the highest were observed in the case of BFRP bars—approx. 2.5%.

### 3.2. The Results of Shear Strength Testing

**Figure 12:**
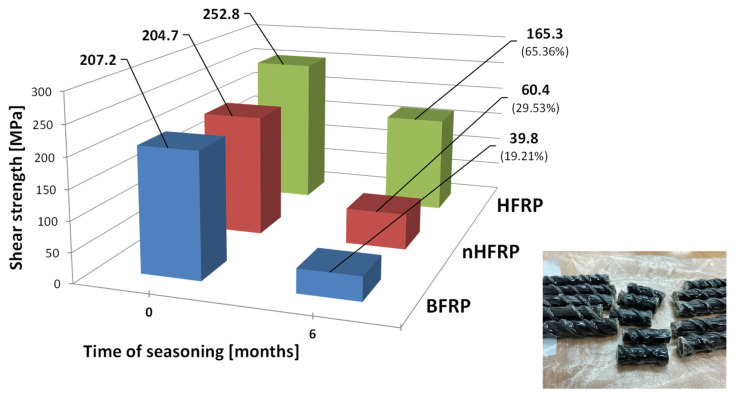
Decrease in shear strength for BFRP, HFRP, and nHFRP bars depending on the seasoning period and typical failure mode.

The shear strength of the BFRP, HFRP, and nHFRP bars before and after six months of seasoning was determined, and then its decrease was estimated. Figure 12 shows the shear strength as a function of the seasoning period and FRP type and the common failure modes. In the initial state (before seasoning), the highest shear strength, 252.8 MPa, was obtained for HFRP bars, followed by 207.2 MPa and 204.7 MPa for BFRP and nHFRP, respectively. The average shear strengths of the bars after 6 months of seasoning were 165.3 MPa for HFRP, 39.8 MPa for BFRP, and 60.4 MPa for nHFRP, which represent 65.4%, 19.2%, and 29.5% of the initial shear strength values, respectively.

## 4. Discussion

The main goals of the research presented in this paper were to evaluate the alkali resistance of new BFRP bars developed through cooperation with an industry partner and to investigate how modification of the BFRP bars through hybridization, i.e., partially replacing the basalt fibers with carbon fibers (HFRP bars) and with an additional admixture of resin with nanosilica (nHFRP), could affect their alkali resistance. All types of tested FRP bars, i.e., BFRP, HFRP, and nHFRP, were produced on the same technological line, using the same basalt and carbon rovings as well as resin type.

**Figure 13:**
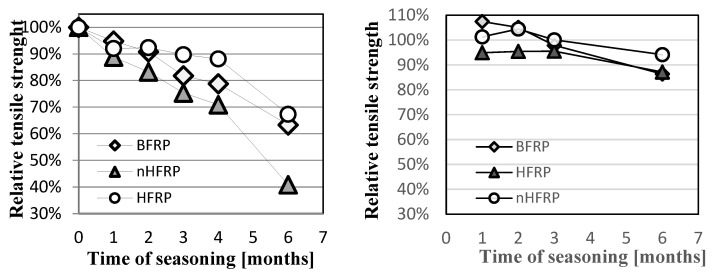
Relative decrease in the strength of bars in the procedure depending on the time of seasoning: Procedure A at 60 °C (**left**) and Procedure B at 23 °C (**right**).

The obtained results of the alkali resistance tests allow for the conclusion that the partial substitution of basalt fibers with carbon fibers (up to 20%) improved not only mechanical properties but also alkali resistance in the case of HFRP. In Procedure A (acc. ACI 440.3R part B6) at 60 °C, the greatest decreases in strength (Figure 13) were noted for bars with nanosilica (nHFRP), and the lowest were found for HFRP bars. After the maximum seasoning period, the BFRP, HFRP, and nHFRP bars reached levels of 60%, 65%, and 41% of initial tensile strength, respectively. In Procedure B (acc. ACI 440.3R part B6), when the seasoning temperature was only 23 °C, the decrease in tensile strength was significantly lower at 10–14%. This indicates that temperature during seasoning is an important factor affecting the alkali resistance evaluation.

Modification with nanosilica (nHFRP bars) did not result in the expected improvement, especially an increase the stability of mechanical properties at elevated temperature. Note that the manufacturing process of nHFPR bars is more difficult (and more expensive) than that of HFRP bars as it requires additional steps, e.g., homogenization of nanosilica particle distribution in the resin binder, which incurs additional cost. The results for nHFRP indicate the difficulty of dispersing nanosilica homogeneously (compare the FRP microstructure cross-sections presented in Table 4).

Using the same procedure, the microstructure cross-sections of the reference HFRP and nHFRP bars were examined after shear testing. Example images of their microstructures are presented in Table 6. The microstructures for both HFRP and nHFRP showed similar images in terms of failures. Cracks propagated through the both basalt fiber and carbon fibers areas and it appears that adhesion in the contact interface between both areas is crucial. In the case of the nHFRP bars, the presence of coarser nanosilica particles and porous areas around the particles is visible. However, it is difficult to claim that these locations are sources of cracks. The nHFRP bars had both the lowest tensile strength after 6 months of seasoning according to Procedure A and the lowest mass loss. This phenomenon indicates that nHFRP bars, due to imperfections in their microstructure, are more easily infiltrated by alkaline solution than other types of FRP bars. This was also the reason why the industry partner ceased the production of nHFRP bars.

**Figure 14:**
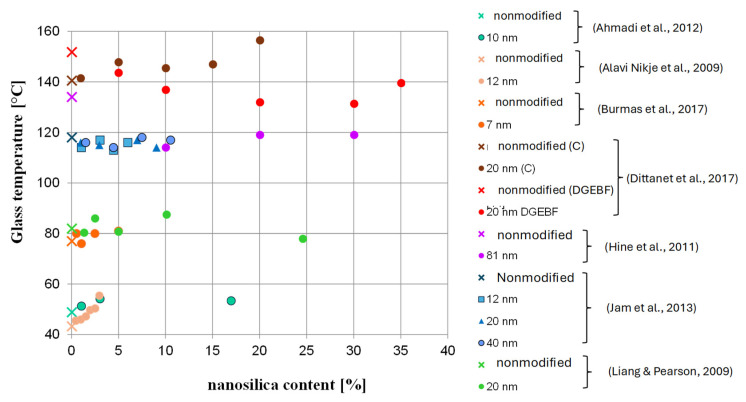
Change in the glass transition temperature with nanosilica content based on literature data [39,40,41,42,43,44,45].

**Figure 15:**
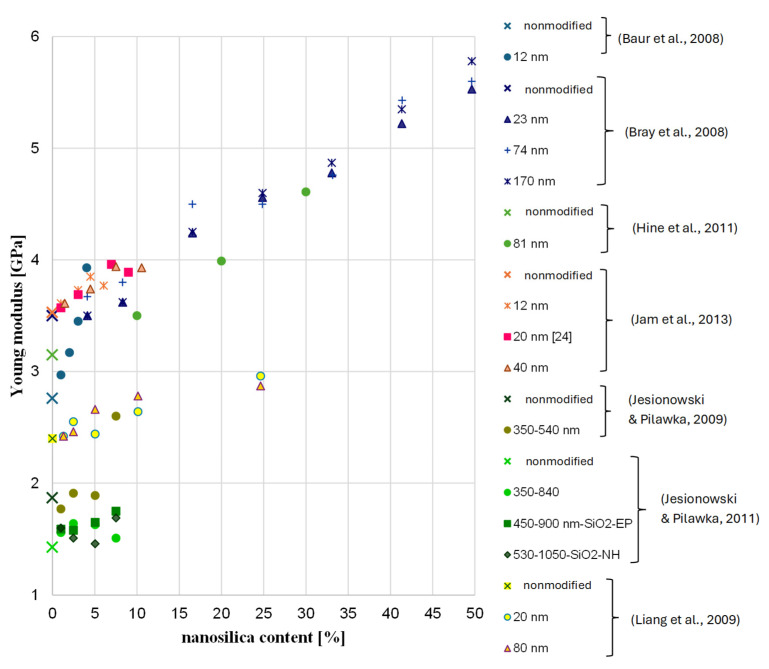
Change in Young’s modulus with nanosilica content based on literature data [43,44,45,46,47,48,49].

**Figure 16:**
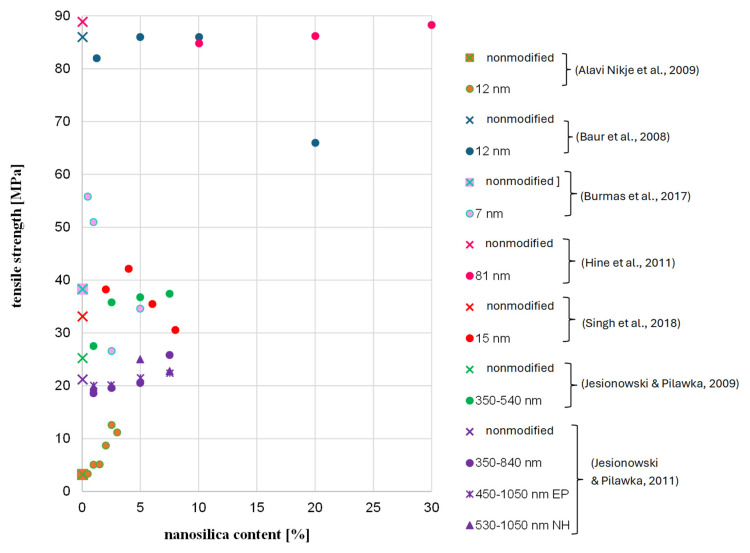
Change in the tensile strength with nanosilica content based on literature data [40,41,43,46,48,49,50].

As for the temperature, although the reason for seasoning samples at 60 °C is to accelerate the degradation process, it is important to not exceed a certain temperature level for FRP bars. By raising the temperature to close to the glass temperature, or even the softening temperature, of “building” epoxy resins, which are commonly used as a matrix, there is a risk of changing the microstructure of the FRP bar; this would impede the testing of durability in context of alkali resistance as a result of changes in the mechanical properties of the resin at the elevated temperature [33,37]. For example, Chiara et al. [38] showed on the basis of literature data that the tensile strength of FRP bars can significantly decrease at temperatures below 60 °C—in some cases, the level of tensile strength retention even reached 80% after exposure of an FRP composite to a temperature 60 °C for 120 min. In turn, Ogrodowska et al. [37] showed that the increase in seasoning time from 30 min to 120 min at 80 °C (no alkaline solution) cased a relatively small decrease in the shear strength of tested FRP bar types—up to 10%—but time to failure decreased by about 50%. Frigione and Lettieri M. [33] concluded that attention should be given to the site temperature when using cold-cured resins: the maximum temperature under working conditions should be at least 20 °C below the expected glass transition temperature of the resin. This suggests that the effect of seasoning FRP in an alkaline solution at 60° did not only correspond to the alkaline solution. Figure 14 shows the changes in the glass temperature of several epoxy resins modified with nanosilica. Based on the results, the effect of nanosilica on glass temperature is not clear. The increase in glass temperature after modification was observed in approximately 50% of modified epoxy resins, especially for binders and hardeners that are cycloaliphatic compounds. In the case of binders based on bisphenol A or F, a decrease in Tg is noted. A more visible advantage of modification was found for Young’s modulus (Figure 15) and partially in the case of the tensile strength (Figure 16). In the case of tested HFRP bars, the modification of epoxy resin with nanosilica (nHFRP) decreased both Young’s modulus and the tensile strength; as a result, the manufacturer developed a new epoxy system for pultrusion with a higher glass temperature (system 1301).

In reality, reinforcing bars will only be operating at higher temperatures for a short period during the concrete setting process, which is simulated in ACI Procedure C. In normal operation (as opposed to special purpose construction or fire conditions), it is unlikely that a temperature of 60 °C will be reached in the vicinity of reinforcing bars. Protchenko and Urbański (2020) investigated beams reinforced with the same types of FRP bars that were subjected to specific fire conditions [51]. The beams reinforced with BFRP bars were destroyed by reinforcement failure, while those reinforced with hybrid FRP bars were destroyed by concrete crushing. When the clear cover of concrete was removed, it was found that the matrix of the HFRP and nHFRP bars had disappeared; however, a large part of the basalt and carbon fibers remained in the same place and continued to withstand the load. The beams reinforced with nHFRP bars failed faster than those reinforced with HFRP and BFRP beams, which was explained by improper execution and incorrect redistribution of nanosilica particles in the nHFRP bars.

The shear strength test was performed to verify its potential usability for the assessment of alkali resistance of FRP bars. Since the preparation of samples and execution of shear tests involve considerably less effort and fewer resources (many times smaller specimens, smaller testing machine), such a method could serve not only to determine the durability of FRP bars as a standardized alternative procedure but also for quality control during production. The basic issue is that FRP bars are anisotropic composites. During tensile strength tests, the mechanical properties of FRP bars depends on the properties of the fibers that carry the load. In the case of shear tests, the strength depends on the properties of fibers in the perpendicular direction, the resin matrix, adhesion between compounds, and eventual defects present inside the bars, e.g., porosity. This is visible when tensile properties are compared with the shear strength in the reference state (before seasoning in an alkaline solution). The tensile strength and the elastic modulus for BFRP, HFRPm and nHFRP are different, e.g., the tensile strength for BFRP was 15% lower than that for nHFRP, while the values of the shear strength were practically the same for both types of FRP bars. The highest results were obtained for HFRP bars in both cases. Therefore, it is difficult to explain the significant decrease in shear strength in the case of BFRP bars after 6 months of seasoning in alkaline solution. SEM observations did not indicate a significant difference in the quality of the microstructures of the BFRP, HFRP, and nHFRP bars. One of the explanations for this could be the specimen preparation method prior to seasoning according to Procedure A. The specimens were not protected at the ends, i.e., the cross-section of the bar was exposed to an aggressive alkaline solution and high temperature. This may be why the shear strength loss is greater than the tensile strength loss for all FRP bar types tested; there is a possibility that the alkali solution migrated along the fibers into the load-applied area and weakened the cross-section. It also indicates the need to protect the FRP sample when considering shear testing to assess alkali (or other chemicals) resistance.

## 5. Conclusions

Based on the alkali resistance tests carried out on the BFRP, HFRP, and nHFRP bars, the following conclusions can be drawn:

1. Modification of the composition of BFRP composite bars through their hybridization, which consisted of the partial replacement of basalt fibers with carbon fibers (HFRP bars), did not decrease the resistance of the bars to the alkaline environment; the decrease in tensile strength after 6 months of seasoning was about 5% lower than in the case of nonmodified BFRP bars.
2. Additional modification of the composition of HFRP composite bars through the addition of nanosilica to the epoxy binder (nHFRP bars) did not provide the expected stability of tensile properties in alkaline solution at elevated temperatures. After 6 months of seasoning at 60°, the decrease in strength was significantly greater (about 40%) than the tensile strength of the BFRP and HFRP bars. SEM observations indicated the difficulty of dispersing nanosilica homogeneously without additional technological operation.
3. The seasoning temperature had a significant effect on the decrease in the tensile strength of FRP bars. The seasoning of BFRP specimens, as well as HFRP and nHFRP specimens, according to ACI Procedure B for 6 months at 23 °C did not result in significant changes in tensile strength, in contrast to the results obtained with Procedure A, where a seasoning temperature of 60 °C was used.
4. The device proposed for maintaining constant strain during the seasoning of the specimens can significantly reduce the cost of conducting tests in which longitudinal elements must be subjected to constant strain over a long period. The results justified its usability for testing the alkali resistance of FRP bars according to ACI Procedure B. It allows alkali resistance testing to be performed at ambient temperature in the present stage, but it can also be developed for testing at 60 °C as recommended by the ACI.
5. The test results of the concreted FRP bar specimens (Procedure C) were practically similar to those obtained with Procedure B. Therefore, this procedure might be used instead of Procedure C to test the actions of alkaline environments and loading simultaneously.
6. The results of the shear strength tests according to Procedure A indicate that the decreases in shear strength were around 5% and 12% greater in comparison to the tensile strength testing for HFRP and nHFRP, respectively. In the case of BFRP bars, the decrease in the shear strength was significantly greater than that in the tensile strength, about 40%.
7. The shear strength test can be employed to determine the durability of FRP bars as a standardized alternative procedure. It is also demonstrated that specimens seasoned prior to the shear strength test should be protected at the ends (e.g., using epoxy resin) to avoid uncontrolled penetration of the alkaline environment along the fibers.

## Figures and Tables

**Table 1:** Mechanical properties of FRP bars (based on [4]).

Property	Steel	GFRP	CFRP	AFRP	BRFP
Nominal yield stress, MPa	40–75	N/A	N/A	N/A	N/A
Tensile strength, MPa	483–690	483–1600	600–3690	1720–2540	1035–1650
Elasticity modulus, GPa	200	35–51	120–580	41–125	25–59
Strain at rupture, %	6–12	1.2–3.1	0.5–1.7	1.9–4.4	1.6–3.0

**Table 2:** The results of alkali resistance tests for various FRP bars.

No.	Authors (Year of Publication)	Material	pH	Seasoning Environment	Temp. [°C]	Duration [Days]	Stress During Seasoning (%)	Retention Ratio (%)
1.	Micelli, Myers, Nanni (2001) [24]	GFRP	12.6	(0.16%)Ca(OH)_2_ + (1%)NaOH + (1.4%)KOH	60	21	no data	<59
		CFRP				42		<92
2.	Benmokrane et al. (2005) [25]	GFRP	12.8	1185 g Ca(OH)_2_, 9 g NaOH, 42 g KOH, 10 L H_2_O	20	420	19÷29	85
					55	30		98
					57	120		83
					61	60		84
					64	60		88
3.	Chen et al. (2007) [18]	GFRP	13.6	2.4 g/L NaOH, 19.6 g/L KOH, 2 g/L Ca(OH)_2_	20	120	no data	86
					40	70		89
					60	70		64
			12.7	0.6 g/L NaOH, 1.4 g/L KOH, 0.037 g/L Ca(OH)_2_	20	120	no data	92
					40	70		92
					60	70		73
4.	Kim et al. (2008) [26]	GFRP	13	(1.4%)KOH, (1%)NaOH (0.16%)Ca(OH)_2_	25	30	no data	75.5
					40			78.3
					80			63.9
					25	60	no data	67.7
					40			70.4
					80			60.3
					25	75	no data	66
					40			67.3
5.	Won et al. (2008) [14]	GFRP	12.6	(1.4%)KOH, (1%)NaOH (0.16%)Ca(OH)_2_	20	300	-	80
					40			70
					60			70
					80			35
6.	Wang Z. et al. (2017) [27]	GFRP	12.7	0.6 g/L NaOH, 1.4 g/L KOH, 0.037 g/L Ca(OH)_2_, 35 g/L NaCl	25	21	20	93.4
						42		93.7
						63		90.7
					40	21	20	86.8
						42		82
						63		74.1
					55	21	20	68.7
						42		73.4
						63		58.6
					25	21	30	88.3
						42		84.3
						63		84.8
					25	21	40	78.9
						42		79.2
						63		68
					40	21	40	Rupture
		BFRP	12.7	0.6 g/L NaOH, 1.4 g/L KOH, 0.037 g/L Ca(OH)_2_, 35 g/L NaCl	25	21	20	92.4
						42		94
						63		92.9
					40	21	20	88.4
						42		89.8
						63		81.7
					55	21	20	69.6
						42		59.8
						63		55.1
					25	21	30	83.1
						42		75.4
						63		78.9
					25	21	40	59.2
						42		54
						63		43.2
					40	21	40	Rupture

**Table 3:** Average value of mechanical properties of tested FRP bars of [MPa] and coefficient of variation [%] determined before seasoning in alkaline environment (acc. [13]).

Property	BFRP	HFRP	nHFRP
Tensile strength [MPa]	1195.57 (2.29)	1538.55 (4.54)	1418.71 (3.77)
Elasticity modulus [GPa]	43.87 (1.96)	73.89 (4.15)	71.00 (1.30)
Elongation at rupture [%]	2.52 (1.98)	1.73 (4.33)	1.72 (2.99)
Diameter [mm]	8.32 (0.30)	8.77 (0.59)	8.61 (0.58)

**Table 4:** Examples of the microstructure cross-sections of the tested BFRP, HFRP, and nHFRP bars at different magnifications (SEM, BSD mode).

**BFRP**	
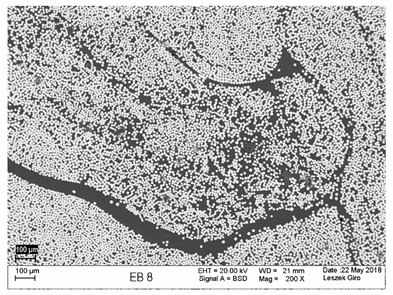	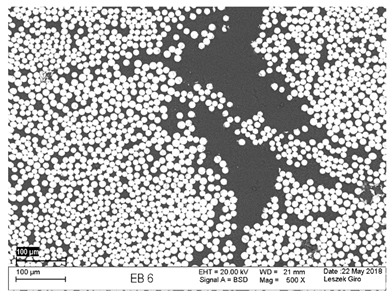
**HFRP**	
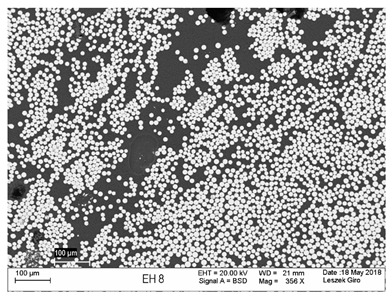	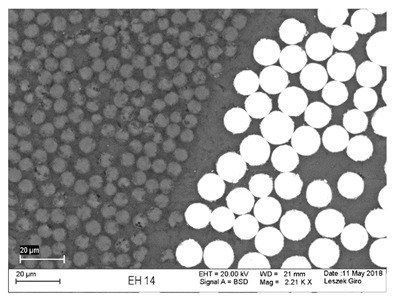
**nHFRP**	
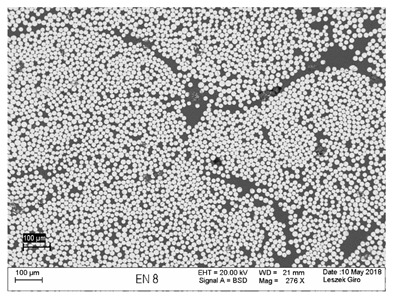	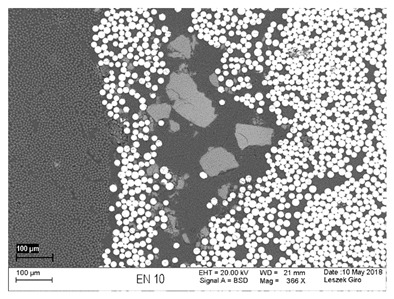

**Table 5:** Mean tensile strength [MPa] and in brackets coefficient of variation [%] (ratio of standard deviation to mean value) of the tested FRP bars depending on the alkali resistance test procedure.

Time of Seasoning [Month]					
	1	2	3	4	6
Procedure A					
BFRP	1133.82 (2.19)	1085.52 (1.68)	977.85 (3.23)	942.22 (5.49)	756.86 (2.66)
HFRP	1415.95 (0.61)	1421.33 (5.45)	1380.51 (5.76)	1355.68 (4.34)	1035.70 (26.77)
nHFRP	1260.35 (15.16)	1181.05 (8.18)	1068.74 (12.28)	1005.78 (6.80)	579.48 (51.54)
Procedure B					
BFRP	1284.78 (1.58)	1256.00 (1.70)	1169.92 (8.73)	-	1032.04 (1.05)
HFRP	1459.98 (4.38)	1468.07 (3.89)	1469.80 (1.33)	-	1340.52 (8.90)
nHFRP	1437.17 (1.36)	1482.13 (1.32)	1419.66 (2.08)	-	1335.71 (10.85)
Procedure C					
BFRP	1270.592 (1.96)	1218.20 (1.12)	1185.17 (1.41)	-	-
HFRP	1461.57 (3.04)	1431.07 (2.62)	1453.08 (2.59)	-	-
nHFRP	1382.66 (-)	1435.98 (3.55)	1394.06 (3.14)	-	-

**Table 6:** Examples of the cross-sectional microstructures of tested HFRP (left) and nHFRP (right) bars after shear test of specimens after seasoning for 6 months (SEM, BSD mode).

**HFRP**	**nHFRP**
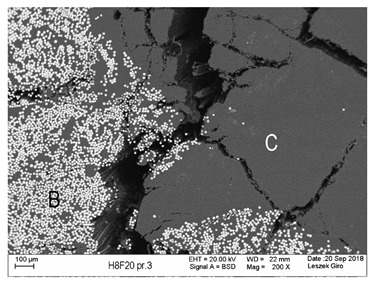	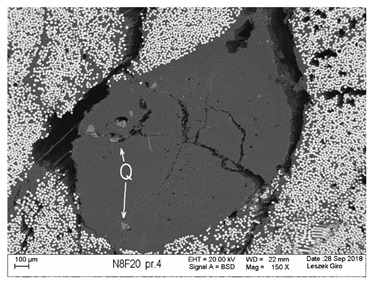
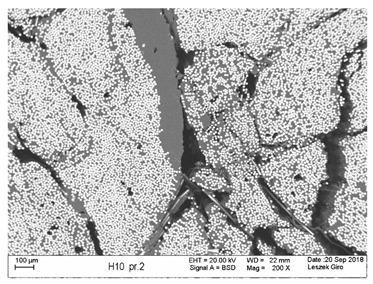	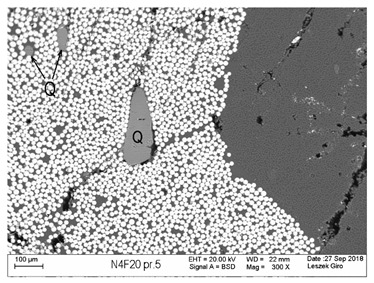
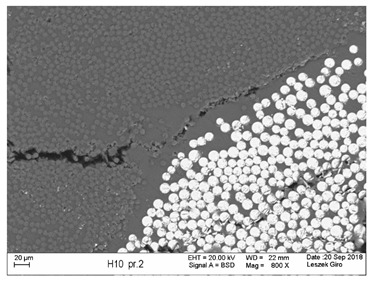	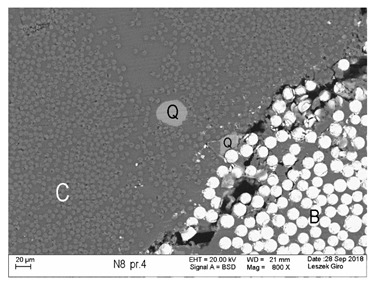

## Data Availability

The original contributions presented in this study are included in the article. Further inquiries can be directed to the corresponding author.
